# Tissue microarrays analysis in chondrosarcomas: light microscopy, immunohistochemistry and xenograft study

**DOI:** 10.1186/1746-1596-3-S1-S25

**Published:** 2008-07-15

**Authors:** Isidro Machado, Francisco Giner, Empar Mayordomo, Carmen Carda, Samuel Navarro, Antonio Llombart-Bosch

**Affiliations:** 1 Department of Pathology, University of Valencia, Valencia, Spain

## Abstract

**Background:**

Chondrosarcoma (Chs) is the third most frequent primary malignant tumour of bone and can be primary or secondary, the latter results mainly from the malignant transformation of a benign pre-existing tumour.

**Methods:**

All the cases diagnosed as Chs (primary tumours, recurrences and/or metastasis and xenotransplanted Chs) from the files of our Department were collected. Only cases with paraffin blocks available were selected (Total 32 cases). Six Tissue Microarrays (TMAs) were performed and all the cases and biopsies were distributed into the following groups: a) only paraffin block available from primary and/or metastatic tumours (3 TMAs), b) paraffin block available from primary and/or metastatic tumours as well as from the corresponding Nude mice xenotransplant (2 TMAs), c) only paraffin block available from xenotransplanted Chs (1 TMA). A reclassification of all the cases was performed; in addition, conventional hematoxylin-eosin as well as immunohistochemistry staining (S100, SOX-9, Ki-67, BCL-2, p53, p16, CK, CD99, Survivin and Caveolin) was analyzed in all the TMA.

**Results:**

The distribution of the cases according to the histopathological pattern and the location of tumours were as follows: fourteen Grade I Chs (all primaries), two primary Grade II Chs, ten Grade III Chs (all primaries), five dedifferentiated Chs (four primaries and one primary with metastasis), and two Chs from cell cultures (Ch grade III). One recurrent extraskeletal myxoid Chs was included as a control in the TMA. Although there was heterogeneity in immunohistochemistry results of the different material analyzed, S100, SOX-9, Caveolin and Survivin were more expressed. The number of passages in xenotransplants fluctuated between 1 and 13. Curiously, in Grade I Chs, these implanted tumours hardly grew, and the number of passages did not exceed one.

**Conclusion:**

The study of Chs by means of TMA techniques is very important because it will improve the assessment of different antibodies applied in the immunohistochemical assays. Xenotransplanted tumours in TMA improve knowledge concerning the variability in the morphological pattern shown by these tumours during the evolution in nudes.

## Introduction

The development of Tissue Microarray (TMA) technology offers the advantage to screening large tissue cohorts for biomarker expression and to examine serial sections obtained from the same tumour specimen and xenograft tumours [1-8]. TMAs are especially suitable for the reproduction of morphological and immunohistochemical (IHC) results in different laboratories [2,4,5]. Chondrosarcoma (Chs) is the third most frequent primary malignant tumour of bone exceeded only by myeloma and osteosarcoma. It is characterised by the production of cartilage and can be primary or secondary, the latter results mainly from the malignant transformation of benign pre-existing tumours [9,10]. Several clinical and histopathological subtypes are recognized and the final diagnoses involve a large group of physicians including pathologists, radiologists and surgeons [10-12]. The conventional Chs can be classified according the location in bone in two groups: central Chs; located inside the medullar cavity and the peripheral Chs; located in the surface of the bone [9]. The histopathology is quite similar in both locations and is classified in three grades: Grade I, II and III. Grade I Chs are characterized by cells with small densely straining nuclei and chondroid or myxoid background, in Grade II Chs the nucleus are of moderate size with increased cellularity and low mitotic rate, Grade III Chs reveal large nuclei in areas with increased cellularity with moderate mitotic rate and scant chondroid or myxoid matrix[9]. Mesenchymal Ch, dedifferentiated Ch and clear cell Ch are described as infrequent variants of Chs and the clinical presentation, radiological findings and histopathological features are different to conventional forms [9-12]. The differential diagnosis is quite difficult between low grade Chs and benign chondral conditions, but usually the clinical data, radiography and morphological picture defines a definitive diagnosis [9,10].

Cytogenetic studies show heterogeneity related to karyotypic complexity, nevertheless, alterations in p16 tumour suppressor gene and p53 are related with Ch progression [9,10,13,14].

Xenograft models in bone tumour are of great importance because nude tumours constitute an easy source of material for tumour characterization using histopathological, IHC, electron microscopy, cytogenetic and molecular biology criteria [6,7,15]. Usually the histology of the tumours is preserved and they can also be followed over subsequent generations in various tumours passages [7,15]. Xenograft models in Chs are performed infrequently and being followed for successive generations is even more sporadic. Few publications exist on TMA study in Chs and the combination with a xenograft study is even more unusual. Therefore, the aims of this study are firstly the analysis of heterogeneity in Chondrosarcomas by means of TMA technology using histopathological and IHC criteria and secondly to analyze their successive xenografts after xenotransplantation into nude mice followed for several passages to describe the histopathological and IHC pattern variations occurring in the original tumour and their successive xenotransplants.

## Materials and methods

### Samples sources

All the cases diagnosed as Chs (primary tumours, recurrences and/or metastasis and xenotransplanted Chs) from the files of our Department were collected. Only cases with paraffin blocks available were selected. All the clinical data of the patients were reviewed. (Total: 32 cases). A reclassification of all the cases according to the new criteria for Chs diagnosis (WHO) was performed.

### Assembly of TMA

Six TMAs were performed and all the cases and biopsies were distributed into the following groups: a) only paraffin block available from primary and/or metastatic tumours (3 TMA), b) paraffin block available from primary and/or metastatic tumours as well as from the corresponding Nude mice xenotransplant (2 TMA), c) only paraffin block available from xenotransplanted Chs (1 TMA). A hematoxylin and eosin stained (H/E) section from each primary tumour and xenograft was prepared and areas of representative non-necrotic neoplasm circled on coverslip. The TMAs were assembled using a manual tissue arrayer (Beecher Instruments, Sun Prairie, WI). Normally, two cores (1 mm in thickness) of each biopsy were performed; nevertheless, more than 2 cores were made if the biopsy revealed a different pattern. All TMAs included two cores of normal kidney or liver as control tissues. Following TMA construction, H/E stained section of the TMA recipient block was prepared and reviewed to confirm the presence of intact neoplasm. Several sections of 5 μ were prepared in order to perform H/E stain as well as different IHC staining.

### Immunohistochemical analysis

IHC analysis was performed using anti-CD99 antibody (clone 12E7, DakoCytomation) at a 1:50 dilution, anti-S100 polyclonal antibody (DakoCytomation) at a 1:200 dilution, anti-SOX-9 polyclonal antibody (Santa Cruz Biotechnology, Santa Cruz, CA) at 1:100 dilution, anti-survivin polyclonal antibody (Santa Cruz Biotechnology, Santa Cruz, CA) at 1: 50 dilution, anti-p16 antibody (clone F12, Santa Cruz Biotechnology, Santa Cruz, CA) at 1:100 dilution, anti-p53 antibody (clone DO7, Novocastra) at 1:50 dilution, pan-CK (AE1/AE3) antibody (DakoCytomation) at 1:50 dilution, anti-Ki-67 antibody (MIB-1, DakoCytomation) at 1:50 dilution, anti-Caveolin (CAV) polyclonal antibody (Santa Cruz Biotechnology, Santa Cruz, CA) at 1:200 dilution, anti-Bcl-2 antibody (clone 124, Novocastra) at 1:50 dilution. Antigen retrieval was performed by pressure cooker boiling for 3 minutes in 10 mmol/L of citrate buffer (pH 6.0). The LSAB method (DakoCytomation) was used, followed by revelation with 3,3'-diaminobenzidine. Cytoplasmic and/or membrane staining was considered positive for CD99, S100, CK, CAV and Bcl-2 antibodies, nuclear staining was considered positive for SOX-9, Survivin, p53, p16 and Ki-67 antibodies. Sections were examined and immunoreactivity was defined as follow: negative, fewer than 5% of tumour cells stained; poorly positive (+), 5% to 10% of tumours cells stained; moderately positive (++), 10% to 50% of tumours cells stained and strongly positive (+++), more than 50% of the tumours cells were stained. All sections were evaluated independently by 3 pathologists (IM, SN and ALLB). The agreement of staining intensity scoring by all was recorded, and in cases of disagreement, intensity and score was determined by consensus.

### Xenotransplant

Male nude mice, were purchase from IFFA-CREDO (Lyon, France), kept under specific pathogen-free conditions throughout the experiment, and provided with vinyl isolates plus sterilized food, water, cage and bedding. The specimens for xenotransplant were obtained at surgery (OT) and placed in a culture medium (RPMI 1640) plus antibiotic at 37 ℃ until transplantation, usually 6 hours after surgery. Fragments of non-necrotic tumour, about 3 to 5 mm in size, were transplanted into the subcutaneous tissue in the backs of two nude mice. The new tumour transfers were made by following the same procedure as in the initial xenotransplant and always under highly sterile conditions. Material from different passages was obtained in order to perform all TMAs. Additional material was obtained for electron microscopy, culture, and frozen sections.

## Results

**Figure 1:**
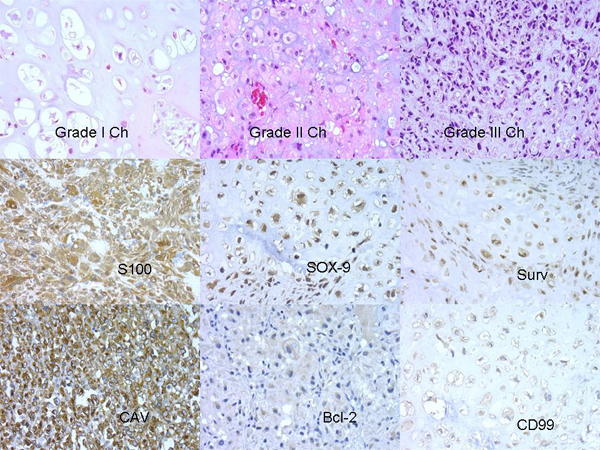
Histopathology and IHC profile in Chs.

Grade I and Grade III Chs were the most frequent histopathological patterns, the distribution of the cases were as follows: fourteen Grade I Chs (all primaries), two primary Grade II Chs, ten Grade III Chs (all primaries) (Figure 1), five dedifferentiated Chs (four primaries and one primary with metastasis) and two Chs from cell cultures (Ch grade III). One recurrent extraskeletal myxoid Chs was included as a control in the TMA. Most of the tumours were located in bone and/or soft tissue. The results of IHC study are given in Table 1. Although there was heterogeneity in IHC results of the different material analyzed, S100, SOX-9, Caveolin and Survivin were more expressed (Figure 1).

**Table 1:** Inmunohistochemical profile. N/A = Not assessable

**Antibodies**	**N/A**	**Negative (-)**	**Poorly positive (+)**	**Moderately positive (++)**	**Strongly positive (+++)**
**S100**	3	0	0	1	28
**SOX-9**	1	2	7	5	17
**Survivin**	2	2	4	3	21
**Caveolin**	1	1	6	12	12
**CD99**	2	8	12	5	5
**p16**	5	10	8	3	6
**p53**	2	16	6	6	2
**Ki-67**	4	7	5	9	7
**Bcl-2**	3	16	7	3	3
**CK**	1	28	2	1	0

**Figure 2:**
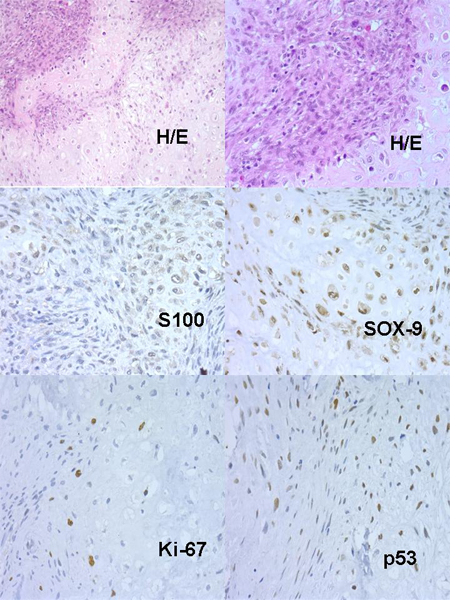
Dedifferentiated Ch, Original Tumour.

**Figure 3:**
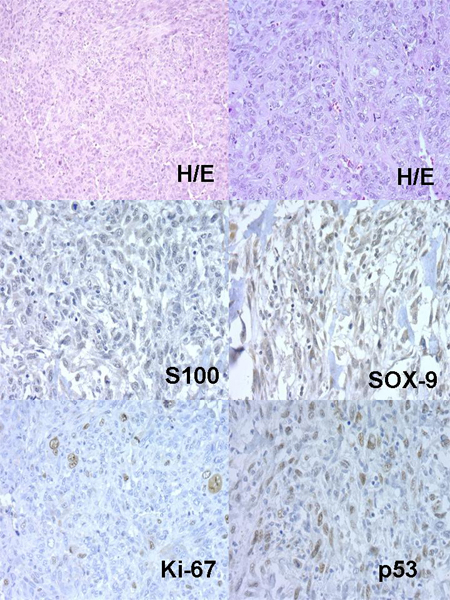
Nude 385 Passage 0.

**Figure 4:**
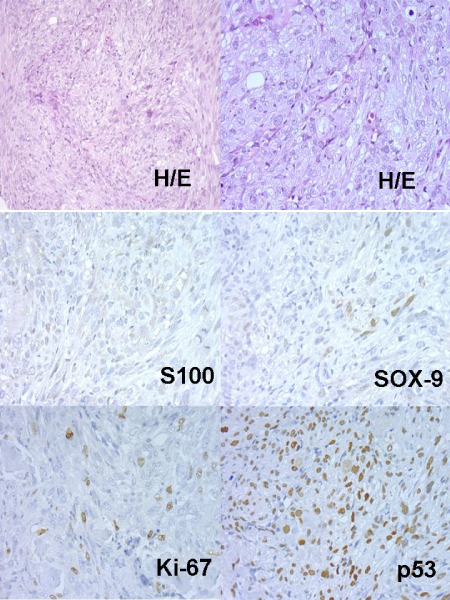
Nude 385 Passage 1.

**Figure 5:**
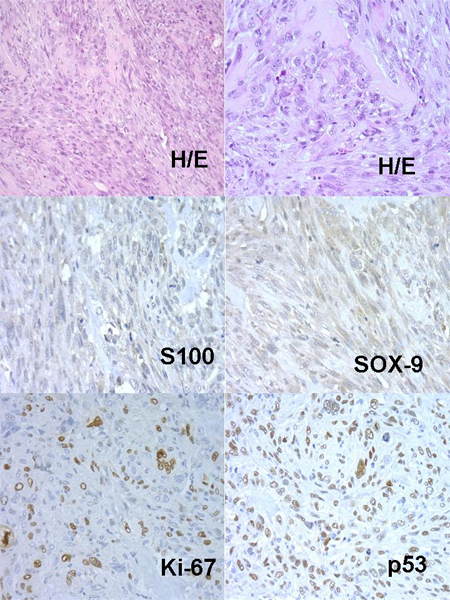
Nude 385 Passage 2.

**Figure 6:**
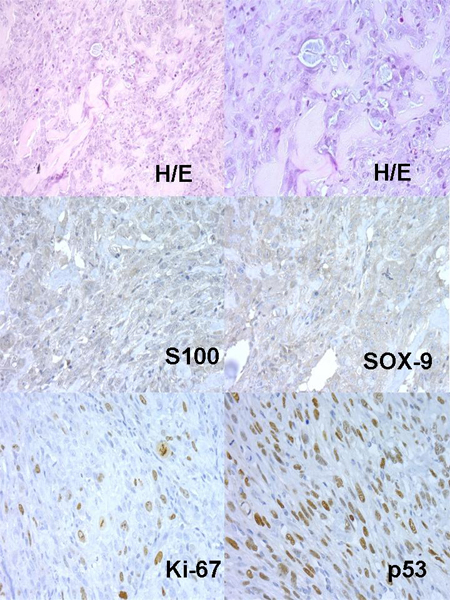
Nude 385 Passage 3.

Concerning xenografts, the number of passages in xenotransplants fluctuated between 1 and 13. Curiously, in low grade – Grade I Chs, these implanted tumours hardly grew, and the number of passages did not exceed one. Nevertheless, in Grade II or Grade III Chs the number of passages were greater. An example of dedifferentiated Chs evolution in nudes is described in Table 2 and (Figures: 2, 3, 4, 5, 6).

**Table 2:** Model of Xenograft in Ch. Evolution in Nude mice

**Material**	**De-differentiation**	**S100**	**SOX-9**	**Ki-67**	**p53**
**Original Tumour**	+	++	+++	+	+
**Nu385-P0**	++	++	++	++	++
**Nu385-P1**	+++	+	+	++	+++
**Nu385-P2**	+++	+	+	+++	+++
**Nu385-P3**	+++ Osteoid	+	+	+++	+++

## Discussion

TMA-based morphological and IHC evaluation of Chs primary tumour and xenograft proved to be a high throughput source in demonstrating the phenotypic variability of original and xenograft Chs. Grading Chs has proven prognostic value, nevertheless the differential diagnosis between low grade Chs and benign chondroid lesions is quite difficult and requires an integration of clinical, radiology and histopathology information [9-12]. Grade I Ch was the most frequent in this cohort, as reported in the literature and most of the tumours appeared in bone and soft tissue [16]. At the histopathological level, the distinction between Grade I, II, III and dedifferentiated Chs was relatively easy using the updated criteria described [9,10]. S100 immunoreactivity in chondroid foci and the transcription factor SOX-9 (regulator of chondrogenesis) has been reported as the most sensitive and confirmatory markers in Chs diagnosis, both were the most expressed in the study. SOX-9 staining differentiates Chs from various small cells round tumour such as small cell osteosarcomas, non-Hodgkin lymphomas and Ewing/PNET family tumours [17]. Recently, the expression of Bcl-2 and parathyroid-like hormone (PTHLH) as two antibodies that supported Ch diagnosis and differentiating them from other benign conditions as osteochondromas has been described [18], although in the present study, Bcl-2 expression was infrequent in the original tumours and in their corresponding xenograft. Curiously, CAV and Survivin were overexpressed in Chs, therefore apoptosis and cell interactions could be related to tumour chondrogenesis. Further studies with larger series could provide new insight into the pathogenesis of Chs.

Xenograft materials are widely used by researchers although in the case of Chs tumours; few studies have been published using TMA technology [19]. As many Chs tumours reveal different patterns in the same biopsy, it is not surprising that the xenograft tumour showed different histopathological pictures in some cases compared with the original tumour. Usually, Grade I Chs showed no variation in histology and IHC between the primary tumours and their xenograft. Intriguingly, in Grade I Chs, these implanted tumours hardly grew, and the number of passages did not exceed one, whereas in Grade III; dedifferentiated Chs as well as in Chs from culture, the numbers of successive passages were higher, and light microscopy as well as IHC study revealed differences between primary tumour and their xenograft. Tumour cells in Chs culture could acquire newer genetic and/or epigenetic alterations that increase the aggressive phenotype; in addition, these cells in culture grow quite fast and replace stromal and necrotic cells from the original tumour. Original tumours (Grade III or dedifferentiated Ch) developed morphological transformation to a poorly differentiated subtype in some of the successive xenotransplanted tumours, in addition differences between IHC expression of chondrogenic differentiation markers (S100, SOX-9), cell cycle regulators (p53, p16) and proliferation marker (Ki-67) were detected. The dedifferentiated Ch xenograft model displayed a morphological transition over subsequent passages in nude mice, initially the tumour showed a biphasic pattern characterized by areas with typical chondroblastic differentiation surrounding by areas with marked dedifferentiation. Over the successive passages, the tumour acquired more dedifferentiation and the last tumours passages revealed osteoid material similar to osteosarcoma. The approximate period between passages was three months. In addition, S100 and SOX-9 expression showed relatively decreased expression and Ki-67, p53 and p16 revealed increased expression in the successive xenograft. Some of these changes may represent newer genetic and/or epigenetic alterations developed in more aggressive subclones or could be induced by the establishment of the xenograft as occurs in tumour progression[6,7]. TMAs in xenografted tumours offer an invaluable tool for performing further research in order to study newer markers of cell differentiation, activation, genetics and cell signalling in Chs. Despite the increasing use of TMAs, limitations remain, particularly in the case of tumours, due to intratumoral heterogeneity of protein expression [1,5]. Therefore in order to ensure TMA representativeness, more representative areas of sections of the original donor block should be selected, as well as increasing the number of cores collected and the size of the single cores [5]. Nowadays, TMAs are becoming an indispensable tool in the study of cancer progression and provide newer insights concerning the biology of several tumours.

## Conclusion

The analysis in TMA of Chs xenograft followed for successive generations provides new information concerning the biology and morphology of these tumours. The histopathology remained similar to the original tumour in most of the cases, but occasionally, as in the model displayed, the tumours acquire more dedifferentiation after several transfer. IHC studies using TMA techniques are useful for the assessment of the antibodies related to Chs.
